# Gene Expression Changes in the Septum: Possible Implications for MicroRNAs in Sculpting the Maternal Brain

**DOI:** 10.1371/journal.pone.0038602

**Published:** 2012-06-06

**Authors:** Changjiu Zhao, Michael C. Saul, Terri Driessen, Stephen C. Gammie

**Affiliations:** 1 Department of Zoology, University of Wisconsin-Madison, Madison, Wisconsin, United States of America; 2 Neuroscience Training Program, University of Wisconsin-Madison, Madison, Wisconsin, United States of America; Max Planck Institute of Psychiatry, Germany

## Abstract

The transition from the non-maternal to the maternal state is characterized by a variety of CNS alterations that support the care of offspring. The septum (including lateral and medial portions) is a brain region previously linked to various emotional and motivational processes, including maternal care. In this study, we used microarrays (PLIER algorithm) to examine gene expression changes in the septum of postpartum mice and employed gene set enrichment analysis (GSEA) to identify possible regulators of altered gene expression. Genes of interest identified as differentially regulated with microarray analysis were validated with quantitative real-time PCR. We found that fatty acid binding protein 7 (Fabp7) and galanin (Gal) were downregulated, whereas insulin-like growth factor binding protein 3 (Igfbp3) was upregulated in postpartum mice compared to virgin females. These genes were previously found to be differentially regulated in other brain regions during lactation. We also identified altered expression of novel genes not previously linked to maternal behavior, but that could play a role in postpartum processes, including glutamate-ammonia ligase (Glul) and somatostatin receptor 1 (Sstr1) (both upregulated in postpartum). Genes implicated in metabolism, cell differentiation, or proliferation also exhibited altered expression. Unexpectedly, enrichment analysis revealed a high number of microRNAs, transcription factors, or conserved binding sites (177 with corrected *P*-value <0.05) that were significantly linked to maternal upregulated genes, while none were linked to downregulated genes. MicroRNAs have been linked to placenta and mammary gland development, but this is the first indication they may also play a key role in sculpting the maternal brain. Together, this study provides new insights into genes (along with possible mechanisms for their regulation) that are involved in septum-mediated adaptations during the postpartum period.

## Introduction

A variety of behavioral, metabolic, structural, and neuroendocrine alterations occur during the transition from a virgin to a lactating state [1]–[4]. These physiological adaptations during lactation are believed to be critically important for the survival and development of the offspring. For example, in rodents, within hours of parturition, the mother retrieves the scattered pups, gathers them together in the nest, and adopts a nursing posture over the pups to permit suckling [5]. Adaptive changes in several neuroendocrine systems during lactation are also evident. For example, the release of both adrenocorticotropic hormone and corticosterone in response to various stressors is markedly suppressed [6], [7]. A striking change in metabolic aspect during lactation is the negative energy balance resulting from the increased energy demand and decreased adaptive thermogenesis [8]. This energy-saving mechanism facilitates the availability of energy for milk production.

The mammalian septum is a heterogeneous forebrain structure that is divided into two main subdivisions, the medial septum (MS) and the lateral septum (LS), and has been linked to various aspects of maternal care, including offspring protection and pup retrieval [9]–[12]. Neuroanatomically, MS is densely interconnected with the hippocampus while LS has extensive reciprocal connections with numerous brain regions known to regulate affect and motivation, such as hypothalamus, amygdala, bed nucleus of the stria terminalis, periaqueductal gray, ventral tegmental area, and raphe nuclei [13]–[15]. Neurochemically, a high number of neurons in LS contain GABA and in subregions of LS, neurons also express other neurotransmitters, such as neurotensin, enkephalin, galanin, and somatostatin [13], [14]. Neurons within MS are predominantly GABAergic and/or cholinergic [16]. LS has long been considered to play a critical role in regulating multiple affective, behavioral and cognitive processes, such as fear, anxiety, depression, aggression, maternal behavior, and social recognition [11], [15], [17]–[19]. MS functions mainly as a region that modulates processes related to attention and memory [20], [21].

Alterations in neuronal activity and gene expression in several brain regions during the postpartum period have been reported. For example, during lactation, neuronal activity of neuropeptide Y (NPY) and agouti-related protein are increased in the arcuate nucleus of hypothalamus (Arc), a site known to be a core feeding center, whereas proopiomelanocortin is reduced [22], [23]. These changes in neuropeptide activity have been proposed to be important in integrating Arc-mediated food intake and energy balance. Expression changes of some genes in forebrain structures during lactation have been demonstrated to be involved in the control of maternal/parental and/or reproductive behaviors [24]–[26]. However, possible alterations in gene expression in the septum that may contribute to the septum-mediated adaptations during lactation remain largely unexplored. In this study, we employed microarray analysis to identify genes in the septum with altered expression during the postpartum period.

As part of this study, we were also interested in how identified gene expression differences may have occurred. Producing the maternal brain is a complex process and is associated with various contributing factors including hormonal changes and sensory input from different events, such as the mating, parturition, nursing, and additional interactions with offspring [27]–[29]. There are likely large numbers of transcription factors that contribute to the gene expression changes but which have not to date been evaluated. Newer data mining tools, such as gene set enrichment analysis (GSEA), provide a unique approach to gain insight into how altered gene expression occurs [30]. The databases include for each gene information on all known binding sites in that region, including those for a wide range of transcription factors and microRNAs (miRNAs), that may be involved in gene expression. MiRNAs are of interest because they have been implicated in peripheral alterations, such as placenta and mammary gland development [31], [32] in the maternal female, but to date no roles for miRNAs have been examined for the maternal brain. In this study, we used GSEA to gain insights into how large scale changes may have been occurring during pregnancy and early lactation with a subset of transcriptional regulators.

## Results

### Genes Identified as being Differentially Expressed in the Septum of Lactating Versus Virgin Mice

Using PLIER analysis with a *P*<0.0025 cutoff value, we identified 116 genes that displayed significant changes in expression (Table 1). Two genes of interest, Gal (*P* = 0.018, fold change is 0.83) and Sstrl (*P* = 0.004, fold change is 1.22) were not included in Table 1 because their *P* values were more than 0.0025. The full list of all 35,557 targets, their relative expression and *P* value ranking using PLIER algorithm is presented in Table S1. The genes identified as being differentially expressed in lactating versus virgin mice were distributed across a number of categories (Table 1). The function/category of each gene was sorted out individually based on the biological process using PubMed and GenBank databases.

**Table 1:** List of genes showing highest significant differences in gene expression between lactating and virgin mice when analyzed with the probe logarithmic intensity error statistics (PLIER) (*P*<0.0025).

Accession#	Gene	Gene title	Fold change
*Apoptosis, anti-apoptosis*			
NM_019735	Apip	APAF1 interacting protein	1.11
NM_026121	Bag4	BCL2-associated athanogene 4	1.17
NM_001033136	Fam82a2	family with sequence similarity 82, member A2	1.07
NM_010477	Hspd1	heat shock protein 1	1.10
NM_026160	Map1lc3b	microtubule-associated protein 1 light chain 3 beta	1.07
*Biosynthesis*			
NM_013490	Chka	choline kinase alpha	1.14
NM_010027	Ddt	D-dopachrome tautomerase	1.16
NM_001033300	Gmps	guanine monophosphate synthetase	1.05
NM_134017	Mat2b	methionine adenosyltransferase II, beta	1.08
*Cell cycle, adhesion, division, death, differentiation and proliferation*			
NM_007591	Calr	calreticulin	1.10
NM_017367	Ccni	cyclin I	1.07
NM_009865	Cdh10	cadherin 10	1.09
NM_009969	Csf2	colony stimulating factor 2 (granulocyte-macrophage)	0.87
NM_019561	Ensa	endosulfine alpha	1.07
NM_023794	Etv5	ets variant gene 5	1.19
NM_021272	Fabp7*	fatty acid binding protein 7	0.69
NM_146001	Hip1	huntingtin interacting protein 1	1.09
NM_008343	Igfbp3*	insulin-like growth factor binding protein 3	1.30
NM_008564	Mcm2	minichromosome maintenance deficient 2 mitotin (S. cerevisiae)	0.91
NM_022889	Pes1	pescadillo homolog 1	1.11
NM_033573	Prcc	papillary renal cell carcinoma (translocation-associated)	1.06
NM_009009	Rad21	RAD21 homolog (S. pombe)	1.08
NM_009082	Rpl29	ribosomal protein L29	0.95
NM_018754	Sfn	stratifin	0.89
NM_028232	Sgol1	shugoshin-like 1 (S. pombe)	0.88
NM_016737	Stip1	stress-induced phosphoprotein 1	1.11
NM_177033	Vwc2	von Willebrand factor C domain containing 2	1.08
*Development*			
NM_016845	Acrbp	proacrosin binding protein	1.10
NM_023598	Arid5b	AT rich interactive domain 5B (MRF1-like)	1.14
NM_008010	Fgfr3	fibroblast growth factor receptor 3	1.15
NM_008494	Lfng	LFNG O-fucosylpeptide 3-beta-N-acetylglucosaminyltransferase	1.13
NM_023248	Sbds	Shwachman-Bodian-Diamond syndrome homolog (human)	1.08
*Metabolism*			
NM_025590	Acot11	acyl-CoA thioesterase 11	1.13
NM_053115	Acox2	acyl-Coenzyme A oxidase 2, branched chain	0.87
NM_021299	Ak3	adenylate kinase 3	1.11
NM_025275	Amz2	archaelysin family metallopeptidase 2	1.10
NM_001038699	Fn3k	fructosamine 3 kinase	1.09
NM_008131	Glul*	glutamate-ammonia ligase (glutamine synthetase)	1.06
NM_028894	Lonrf3	LON peptidase N-terminal domain and ring finger 3	1.24
NR_028353	Nt5c2	5′-nucleotidase, cytosolic II	1.07
NM_028794	Nudt9	nudix (nucleoside diphosphate linked moiety X)-type motif 9	1.09
NM_009787	Pdia4	protein disulfide isomerase associated 4	1.15
NM_027959	Pdia6	protein disulfide isomerase associated 6	1.20
NM_008832	Phka1	phosphorylase kinase alpha 1	1.17
NM_001083110	Pja1	praja1, RING-H2 motif containing	1.08
NM_025882	Pole4	polymerase (DNA-directed), epsilon 4 (p12 subunit)	0.94
NM_011275	Rnaseh1	ribonuclease H1	1.07
NM_025683	Rpe	ribulose-5-phosphate-3-epimerase	1.08
NM_001163571	Senp3	SUMO/sentrin specific peptidase 3	1.10
NM_001177833	Smox	spermine oxidase	1.14
*Phosphorylation, dephosphorylation*			
NM_013557	Eif2ak1	eukaryotic translation initiation factor 2 alpha kinase 1	1.07
NM_001004144	Git1	G protein-coupled receptor kinase-interactor 1	1.06
NM_144843	Mtmr6	myotubularin related protein 6	1.07
NM_001110218	Ppm1h	protein phosphatase 1H (PP2C domain containing)	1.15
NM_027673	Tssk4	testis-specific serine kinase 4	0.91
*Protein folding*			
NM_009838	Cct6a	chaperonin containing Tcp1, subunit 6a (zeta)	1.08
NM_021422	Dnaja4	DnaJ (Hsp40) homolog, subfamily A, member 4	1.17
NM_198899	Uggt1	UDP-glucose glycoprotein glucosyltransferase 1	1.09
*Protein ubiquitination, deubiquitination*			
NM_019927	Arih1	ariadne ubiquitin-conjugating enzyme E2 binding protein homolog 1 (Drosophila)	1.05
NM_133247	Usp33	ubiquitin specific peptidase 33	1.08
*Regulation of transcription*			
NM_017406	Atf6b	activating transcription factor 6 beta	1.08
NM_011714	Baz1b	bromodomain adjacent to zinc finger domain, 1B	1.08
NM_019682	Dynll1	dynein light chain LC8-type 1	1.09
NM_008065	Gabpa	GA repeat binding protein, alpha	1.12
NM_001080817	Prdm10	PR domain containing 10	1.06
NM_027748	Taf3	TAF3 RNA polymerase II, TATA box binding protein (TBP)-associated factor	1.08
NM_001077364	Tsc22d3	TSC22 domain family, member 3	1.34
*RNA-related function*			
NM_026623	Nudt21	nudix (nucleoside diphosphate linked moiety X)-type motif 21	1.13
NM_016813	Nxf1	nuclear RNA export factor 1 homolog (S. cerevisiae)	1.09
NM_026045	Prpf18	PRP18 pre-mRNA processing factor 18 homolog	1.08
NM_027911	Raver1	ribonucleoprotein, PTB-binding 1	0.93
NM_026886	Srrm4	serine/arginine repetitive matrix 4	1.12
NM_198102	Tra2a	transformer 2 alpha homolog (Drosophila)	1.21
*Signal transduction*			
NM_001128084	Arhgap21	Rho GTPase activating protein 21	1.04
NM_011332	Ccl17	chemokine (C-C motif) ligand 17	0.83
NM_009895	Cish	cytokine inducible SH2-containing protein	1.13
NM_010172	F7	coagulation factor VII	0.89
NM_181748	Gpr120	G protein-coupled receptor 120	0.89
NM_008630	Mt2	metallothionein 2	0.93
NM_010934	Npy1r *	neuropeptide Y receptor Y1	1.23
NM_027571	P2ry12	purinergic receptor P2Y, G-protein coupled 12	0.92
NM_008882	Plxna2	plexin A2	1.05
NM_009067	Ralbp1	ralA binding protein 1	1.08
NM_001039089	Sel1l	sel-1 suppressor of lin-12-like (C. elegans)	1.07
NM_008507	Sh2b3	SH2B adaptor protein 3	1.05
*Translation*			
NM_013854	Abcf1	ATP-binding cassette, sub-family F (GCN20), member 1	1.07
NM_029735	Eprs	glutamyl-prolyl-tRNA synthetase	1.06
NM_030722	Pum1	pumilio 1 (Drosophila)	1.07
*Transport*			
NM_001122820	Ap3m2	adaptor-related protein complex 3, mu 2 subunit	1.06
NM_025828	Lman2	lectin, mannose-binding 2	1.10
NM_001013374	Lman2l	lectin, mannose-binding 2-like	1.09
NM_019983	Rabgef1	RAB guanine nucleotide exchange factor (GEF) 1	1.08
NM_009199	Slc1a1	solute carrier family 1 (neuronal/epithelial high affinity glutamate transporter, system Xag), member 1	1.12
NM_011394	Slc20a2	solute carrier family 20, member 2	0.92
NM_011400	Slc2a1	solute carrier family 2 (facilitated glucose transporter), member 1	1.13
NM_028064	Slc39a4	solute carrier family 39 (zinc transporter), member 4	0.89
NM_172269	Vps18	vacuolar protein sorting 18 (yeast)	1.06
*Others*			
NM_027907	Agxt2l1	alanine-glyoxylate aminotransferase 2-like 1	1.64
NM_025675	Atpbd4	ATP binding domain 4	0.92
NM_177759	Ccdc60	coiled-coil domain containing 60	0.84
NM_001081345	Chd2	chromodomain helicase DNA binding protein 2	1.08
NM_030137	Cstad	CSA-conditional, T cell activation-dependent protein	0.79
NM_145570	Fam176a	family with sequence similarity 176, member A	1.19
NM_001033440	Gm1587	predicted gene 1587	0.89
NM_001024720	Hmcn1	hemicentin 1	0.85
NM_033322	Lztfl1	leucine zipper transcription factor-like 1	1.09
NM_001102613	Phldb3	pleckstrin homology-like domain, family B, member 3	0.86
NM_027241	Polr3gl	polymerase (RNA) III (DNA directed) polypeptide G like	1.07
NM_175388	Rnf169	ring finger protein 169	1.07
NM_022324	Sdf2l1	stromal cell-derived factor 2-like 1	1.24
NM_183136	Spink8	serine peptidase inhibitor, Kazal type 8	1.22
NM_175106	Tmem177	transmembrane protein 177	1.12
NM_029074	Tmem188	transmembrane protein 188	1.09
NM_153525	Tmem41b	transmembrane protein 41B	1.15
NM_025749	Zfp474	zinc finger protein 474	0.90
NM_146203	Zfp764	zinc finger protein 764	0.90

Fold change greater than 1.0 represents increases, while less than 1.0 indicates decreases in lactating versus virgin mice.

* Indicates gene expression result obtained from microarray analysis was further verified using qPCR.

### Verification of Microarray Results by Quantitative Real-time PCR Analysis

**Figure 1:**
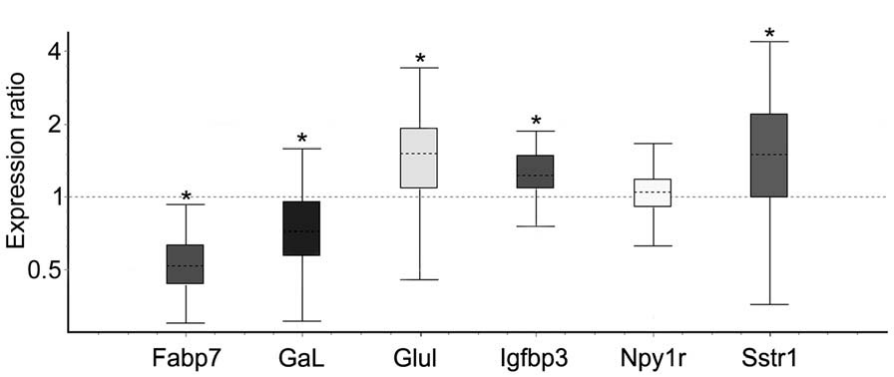
Quantitative real-time PCR analysis of Fabp7, Gal, Glul, Igfbp3, Npy1r and Sstr1 expression in the septum. Relative expression distribution of mRNA (y-axis) represented as a ratio of maternal versus virgin mice, was calculated using Ywhaz and CycA as reference genes, and shown by box-and-whisker plot as medians (dashed lines), interquartile range (boxes) and ranges (whiskers). Ratios over one indicate genes with higher expression in maternal relative to virgin mice, and ratios of less than one indicate genes with lower expression in maternal as opposed to virgin mice. Confirming array results, postpartum mice exhibited increased Glul, Igfbp3, Sstr1and decreased Fabp7 and Gal mRNA levels relative to virgin control mice. Npy1 was not confirmed. *P<0.05 maternal mice versus virgin control.

Consistent with the findings from microarray analysis, mRNA levels of three representative genes, Glul (*P* = 0.023), Igfbp3 (*P* = 0.004) and Sstr1 (*P* = 0.048) were identified as being significantly up-regulated in maternal mice as compared to virgin mice, whereas Fabp7 (*P*<0.001) and Gal (*P* = 0.023) were significantly down-regulated (Fig. 1). While Npy1r mRNA levels were found to be significantly increased in lactating versus virgin mice in the microarray analysis, we did not confirm a difference between the two groups using highly sensitive real-time PCR technique (Fig. 1).

### Gene Set Enrichment Analysis Results

When evaluating genes upregulated in postpartum mice, 177 unique regions involved in transcriptional regulation reached significance (corrected *P*-value <0.05). The majority of these were miRNA binding sites (67%; 119 out of 177). For example, the site with the fifth highest *p*-value was a binding site for miRNA, Mir-183. For some binding sites, multiple miRNAs were listed. For example, for the 10^th^ most significant region is the conserved binding site of AGCACTT, which can be bound by multiple miRNAs, including Mir-93 and Mir-302B. A partial (Table 2) and full list (Table S2) of the GSEA results are provided. The binding sites for 29 different transcription factors were identified as significantly upregulating gene expression in maternal mice (Table 3, Table S2). The database includes target data from multiple species and in some cases a given transcription factor (e.g., YY1) reached significance with multiple species. Here we only counted each transcription factor once and only list those associated with mammals (e.g., mouse, human). Twenty seven additional conserved binding sites in close proximity to genes were also identified as being linked with upregulated expression in maternal mice (Table S2). For these regions, no known transcription factor or miRNA has been identified that target these regions.

**Table 2:** List of all miRNAs found to be significantly linked with upregulated gene expression in postpartum mice using GSEA.

MIR-1	MIR-30E-5P	MIR-138	MIR-195	MIR-301	MIR-381	MIR-501
MIR-9	MIR-31	MIR-139	MIR-197	MIR-302A-D	MIR-382	MIR-505
MIR-15A-B	MIR-32	MIR-141	MIR-199A-B	MIR-320	MIR-409-3P	MIR-512-3P
MIR-16	MIR-33	MIR-142-3P	MIR-200A-C	MIR-323	MIR-409-5P	MIR-512-5P
MIR-17-3P	MIR-34A-C	MIR-142-5P	MIR-202	MIR-324-3P	MIR-410	MIR-515-3P
MIR-17-5P	MIR-92	MIR-143	MIR-203	MIR-326	MIR-422A-B	MIR-516-5P
MIR-18A-B	MIR-93	MIR-144	MIR-204	MIR-329	MIR-424	MIR-518A-2
MIR-19A-B	MIR-96	MIR-145	MIR-205	MIR-335	MIR-429	MIR-518C
MIR-20A-B	MIR-101	MIR-148A-B	MIR-206	MIR-337	MIR-432	MIR-519A-D
MIR-21	MIR-103	MIR-149	MIR-211	MIR-346	MIR-448	MIR-520A-H
MIR-22	MIR-105	MIR-150	MIR-212	MIR-361	MIR-449	MIR-524
MIR-24	MIR-106A-B	MIR-152	MIR-214	MIR-363	MIR-452	MIR-526B
MIR-25	MIR-107	MIR-153	MIR-216	MIR-365	MIR-485-3P	MIR-527
MIR-26A-B	MIR-122A	MIR-154	MIR-218	MIR-367	MIR-485-5P	
MIR-27A-B	MIR-124A	MIR-155	MIR-219	MIR-369-3P	MIR-487	
MIR-28	MIR-125A-B	MIR-181A-D	MIR-221	MIR-372	MIR-493	
MIR-30A-3P	MIR-128A-B	MIR-182	MIR-222	MIR-373	MIR-494	
MIR-30A-5P	MIR-129	MIR-183	MIR-223	MIR-374	MIR-495	
MIR-30B-D	MIR-130A-B	MIR-186	MIR-224	MIR-377	MIR-496	
MIR-30E-3P	MIR-132	MIR-194	MIR-299-5P	MIR-378	MIR-497	

For a given binding site near an upregulated gene, in some cases only one miRNA is identified, but for other sites multiple miRNAs are identified. In the list, if a dash separates two letters, that indicates all miRNAs within those letters were also significant (e.g., MIR-181A-D indicates miRNAs 181A, 181B, 181C, and 181D). Table S2 provides additional information on the binding sites for the miRNAs and p-values.

**Table 3:** List of transcription factors (and corrected *p*-values) that are significantly linked to upregulation of subsets of genes in maternal mice using GSEA.

ELK1	0.0000	RFX1	0.0000	MAX	0.0080	TFDP2	0.0210
YY1	0.0000	GABPB2	0.0010	MYC	0.0080	ERR1	0.0220
NRF2	0.0000	E2F	0.0010	SOX9	0.0080	FOXN1	0.0230
ATF2	0.0000	HSF1	0.0020	HLF	0.0080	PAX3	0.0250
E4F1	0.0000	HIF1A	0.0020	ZF5	0.0090	E2F4	0.0390
E4BP4	0.0000	ATF4	0.0040	HSF2	0.0130		
NFMUE1	0.0000	E2F1	0.0060	NMYC	0.0130		
NRF1	0.0000	SREBF1	0.0070	TFDP1	0.0190		

GSEA website (http://www.broadinstitute.org/gsea/index.jsp) provides information on the conserved binding motif along with all gene targets. Table S2 provides information on *p*-values and names used by GSEA. Abbreviations: ATF, activating transcription factor; E2F, E2F transcription factor; E4F, E4F transcription factor; ELK1, ELK1, member of ETS oncogene family; ERR1, oestrogen receptor related 1; GABPB2, GA repeat binding protein, beta 2; HIF1, hypoxia inducible factor 1; HLF, hepatic leukemia factor; HSF, heat shock factor; MAX, max protein; MYC, myelocytomatosis oncogene; NMYC, neuroblastoma myc-related oncogene; NRF1, nuclear respiratory factor 1; PAX3, paired box gene 3; RFX1, regulatory factor X, 1; SF1, splicing factor 1; SOX9, SRY-box containing gene 9; SREBF1, sterol regulatory element binding transcription factor 1; TFDP, transcription factor Dp; YY1, YY1 transcription factor; ZF5, zinc finger protein 5.

## Discussion

This study used Affymetrix microarray analysis and real-time PCR techniques to examine CNS gene expression changes in the septum during lactation. We identified 116 genes (*P*<0.0025 as analyzed by PLIER) and 2844 genes (using a lower cutoff of *P*<0.05) which were distributed across a number of categories in biological process, as being differentially expressed in lactating versus virgin mice. Some of the genes identified are consistent with previous studies testing gene expression changes during lactation. Our study also found a list of novel genes that may play a role in the transition from a non-parental to a parental state. Further, using data mining approaches and GSEA we identified a number of miRNAs and transcription factors that may play a role in producing the maternal brain.

### Identification of Expression Changes During Lactation Consistent with Previous Studies

In the septum, mRNA of Igfbp3 increased about 30% during lactation (Table 1), and was confirmed by qPCR (Fig. 1). This result is consistent with previous findings that Igfbp3 was increased in the arcuate nucleus of hypothalamus (Arc)/ventromedial nucleus of hypothalamus (VMH) [33], and in large hypothalamic CNS regions in previous maternal array studies [34]. Igfbp3 is a member of the superfamily of insulin-like growth factor (IGF) binding proteins and is expressed in the brain. In vivo studies show that Igfbp3 inhibits IGF action by modulating the bioavailability and distribution of IGFs [35]. One possibility is that increased Igfbp3 in the septum during lactation would result in reduced IGF action, but this would need to be determined. Given that IGF negatively regulates food intake and body weight [36], the increased expression of Igfbp3 would be expected to increase food intake through an IGF-mediated mechanism, consistent with the low levels of IGF in association with the metabolic adaptations that occur during lactation [37]. Based on its role in neurogenesis [38], Igfbp3 has been linked to the involvement in certain disease processes, such as ischemia, hypoxia and autism [39], [40]. There are multiple pathways by which Igfbp3 could possibly interact with maternal circuitry.

Fabp7 transcript was reduced about 30% during lactation in this study and was also found to be significantly decreased in maternal rodents in Arc [33] and hypothalamic CNS regions [34] in previous array studies. The mammalian Fabp7 is expressed in radial glial cells, astrocytes, and neuronal cell precursors, functioning as a modulator of cell differentiation, proliferation, and neurogenesis [41]. Interestingly, Fabp7 was preferentially expressed in numerous astrocytes in the amygdala and septum, regions known to be critically involved in the regulation of fear and anxiety [42]. In addition, previous studies show that this gene modulates the processes of sleep, memory, and anxiety [42], [43]. Several lines of evidence associate Fabp7 with neuropsychiatric diseases such as schizophrenia [44]. Fabp7-deficient mice displayed enhanced fear memory and anxiety in adulthood [42]. Together with the functional significance of the septum in fear, anxiety and memory [15], [20], [21], these findings suggest the possibility that Fabp7 plays an important role in emotional changes that occur during the postpartum period.

We found a decrease of galanin in the gene array, which was confirmed by qPCR. Significant reduction of galanin during lactation has been observed in previous array studies [33], [34]. Galanin is a 29 amino-acid (30 in humans) neuropeptide and is richly expressed in the CNS. Although galanin was not listed in the top identified genes in terms of significance (*P* = 0.018), it was selected as a gene of interest since this peptide has been known to regulate a variety of behavioral processes, including food intake, sexual behavior, learning, memory, reward, cognition, sleep, seizure, as well as emotion/mood-related behaviors, such as stress, fear, anxiety and depression [45]–[47]. Given that LS has many roles in common with galanin in the processes of fear, anxiety and depression, galanin may be an interesting candidate gene for regulation of LS-mediated affective processes.

### Identification of Novel Genes During Lactation

The upregulation of Glul mRNA in the septum of maternal mice was noteworthy for a few reasons. The astrocytic Glul or enzyme glutamine synthetase (GS), is a key enzyme involved in the glutamate-glutamine cycle. Glutamate is metabolized mainly to glutamine by the catalyzation of Glul. Further, glutamine may also be utilized for de novo synthesis of GABA, a major inhibitory neurotransmitter. Thus, Glul modulates levels of glutamate, glutamine, and GABA in the brain [48], and influences GABAergic neurotransmission [49]. As GABA signaling regulates maternal defense [11], it may be that Glul plays a role in the emergence of offspring protection during the postpartum period. Research on the role of excitatory neurotransmitter glutamate in maternal behavior has so far received little attention, but future work evaluating glutamate and maternal care appears to be merited.

We identified increased Sstr1 transcript in the septum during lactation. The neuropeptide, somatostatin, has been linked to hormone release, cell proliferation, and many pathophysiological processes of brain disorders [50]. Somatostatin exerts its actions by interacting with specific G-protein coupled receptors. Sstr1 acts as an inhibitory autoreceptor located on somatostatin neurons and is involved in the modulation of the ultradian release of growth hormone from pituitary [51]. As somatostatin has been shown to regulate GABA receptor functions [52], and GABA signaling is involved in maternal defense, it will be interesting to know whether and how Sstr1 regulates this aspect of maternal care.

It should be noted that many genes implicated in metabolism, cell differentiation, or proliferation exhibited altered expression pattern during lactation, suggesting that septum may be an important neural site for lactation-induced metabolic and structural alterations. As can be seen in Table 1, 18 out of 116 genes were linked to cell division, death, or proliferation, while about 16% were metabolism-related genes. However, the mechanism by which these genes regulate metabolic and structural adaptations during lactation remains to be evaluated. This study provides potential candidate genes for future research to answer these questions.

### Genes Previously Shown to be Differentially Expressed During Lactation Not Identified in the Present Study

Expression of neuropeptide Y, preproenkephalin, and oxytocin has been reported to be increased during lactation [22], [24], [33], [34], whereas proopiomelanocortin was down-regulated [53]. Although these changes were not observed in this study, one possible explanation for negative results is a region-specific effect as these neuropeptides were examined in other brain regions such as hypothalamus and Arc, while the septum was tested in this study. Alternatively, as discussed below, using the whole septum as a target, rather than subregions of septum, may contribute to the lack of difference. In addition to the above two possible factors, time lapse between the parturition and the gene analysis may be another contributing factor. Previous studies have shown that gene expression in specific brain regions undergoes a time-course change from parturition through lactation [24], [54], [55]. For example, preproenkephaline mRNA in the anterior arcuate nucleus of lactating versus virgin females significantly increased on postpartum day 10, but did not differ on postpartum day 3 [24], suggesting that gene expression is dynamic and alters during the postpartum period. Future study will be directed towards understanding the time-course effect on gene expression during the postpartum period.

### Gene Set Enrichment Analysis

One surprising result from this study was the finding of a large number of miRNAs and transcription factors (N = 177) that were significantly linked to the upregulation of genes in maternal mice, whereas no significant links were found with downregulated maternal expression. Why so many regulators for upregulation, but not one for downregulation were found is not clear, but the finding may reflect the underlying processes of developing the maternal brain. It was also surprising to find a high number of miRNAs that were linked to upregulated gene expression. miRNAs are mostly linked with downregulation of gene expression as they can bind to mRNA transcripts, leading to degradation or suppressed translation [56]. Therefore, one explanation could be that in the process of developing the maternal brain, a number of miRNAs are themselves downregulated and this, in turn, leads to elevated expression of select genes. MiRNAs appear to play important roles in mammalian females in both developments of the placenta and mammary glands [31], [32]. Thus, an important role for miRNAs in reproduction has already been documented. To our knowledge, this study is the first to suggest that miRNAs may also play an important role in producing the maternal brain, but direct studies are needed to address this possibility.

The identification of 29 transcription factors, including Elk-1, YY1, and HSF1, that were linked to upregulated genes provides possible new insight into how the maternal brain may be produced. Although key roles for estrogen and progesterone receptors have been found in producing the maternal brain [27], [28], steroid receptors were not identified here, with the exception of estrogen related receptor 1 (Err1). One explanation is that steroid receptors alter expression of other transcription factors and the ones identified here may be key agents for some of the final changes in maternal gene expression in septum. Some of the transcription factors identified by GSEA were also found to have altered expression from the PLIER array analysis (*P*<0.05), such as Hsf1. It is not necessarily surprising that transcription factors or miRNAs linked by GSEA to altered expression are themselves not found to be differentially expressed because their action could have occurred prior to the day of tissue collection (e.g., during pregnancy or early postpartum).

It was also noteworthy that some conserved binding sites were found that were linked to maternally upregulated genes, although there are currently no known factors that bind to these sites. Future studies that determine whether or how these sites are acted upon will be critical for developing an understanding of the relevance of these binding sites.

### Methodological Considerations and Limitations

A number of factors contribute to the formation of the maternal brain, including the experience of mating, pregnancy, parturition, lactation, and the sensory input from the pups. All these factors orchestrate many of changes in gene expression that occur during the postpartum period. This study seeks to explore the results of this constellation of experiences by examining the differences in gene expression between virgin mice, who have never been exposed to any of these experiences, and lactating mice, who have been exposed to all of them. Thus, the observed changes in gene expression in postpartum female mice do not simply reflect maternal care or lactation, but rather the integration of multiple factors. In this study, we did not discriminate between virgins at different stages of the estrus cycle although estrous stage can influence gene expression [57]. Virgin mice were either in estrus, diestrus I and diestrus II (periods of relatively consistent estradiol and progesterone levels), but not proestrus. In this way, only differences large enough to rise above any noise created by varying estrus states appear in the results. While this noise may obscure differences in gene expression, the present approach serves to highlight general differences in virgin and postpartum mice. However, subtler difference between the virgin and lactating mice may be missed. It should be noted that virgin female mice were fed a breeder diet to provide identical food to both groups. This feeding paradigm may have had a somewhat different effect on gene expression changes relative to regular rodent chow as breeder diet differentially modulates expression of genes implicated in food intake and energy balance due to its high fat content [58], [59].

One limitation of using whole septum is that we are unable to determine whether changes occur throughout septum or are specific to subregions. Future studies examining subregions can address this issue. Also, if changes in gene expression occur in opposite directions within these subregions, then the difference may be missed as false negatives. A related general issue is that microarray analysis can produce false negatives. The chips target a subset of regions along a given mRNA transcript and it is possible with different targeting that significant differences may have been found. Although different algorithms produce different false negative rates [60], [61], the approach used here, PLIER, is the current recommended algorithm as it introduces a higher sensitivity to changes in abundance for targets near background [60]. One example of a possible false negative is that we recently glutamic acid decarboxylase (GAD67) mRNA in the septum to be upregulated in lactating versus virgin mice (unpublished observation), while no difference was observed in this study. Microarray technology has become a useful tool for analyzing gene expression profiling and in identifying new genes or molecular pathways involved in multiple processes [62]–[64]. Five out of six genes identified as being differentially expressed (except Npy1r) in this study were confirmed by real-time PCR, validating our microarray analysis as reliable means to evaluate gene expression.

## Conclusions

Using high density oligonucleotide microarrays, we observed a distinct gene expression profile in the septum of maternal mice. Given that the identified genes may play a role in diverse biological processes, this study provides new insights into the genes linked to the septum and brain changes that promote the emergence of maternal care during the postpartum period. Our findings suggest a potential key role for miRNAs in creating the maternal brain and could open an important new area of research. Future studies could involve directly evaluating and manipulating miRNAs to determine the relative importance of miRNAs in producing a maternal brain.

**Figure 2:**
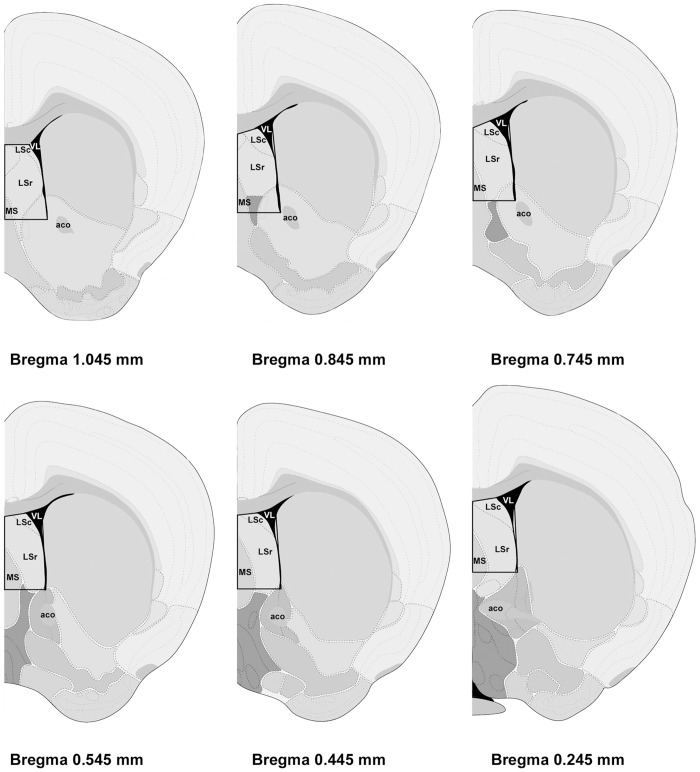
Schematic representation of the brain regions (boxed areas) dissected for gene array analysis. Distance from Bregma in the rostrocaudal planes is indicated. Reprinted and modified from The Allen Mouse Brain Atlas (reference atlas version 1, 2008). Abbreviations: LSc, caudal part of lateral septal nucleus; LSr, rostral part of lateral septal nucleus; MS, medial septal nucleus; VL, lateral ventricle; aco, anterior commissure, olfactory limb.

**Table 4:** Primers of interest and reference genes used for quantitative real-time PCR assay.

Gene	Full name	NCBI accession number		Primer	Primer position
CycA	Cyclophilin A	NM_008907.1	Forward	5′-TGCTGGACCAAACACAAACG-3′	
			Reverse	5′-GCCTTCTTTCACCTTCCCAAA-3′	
Fabp7	Fatty acid binding protein 7	NM_021272.3	Forward	5′-TAAGTCTGTGGTTCGGTTGG-3′	329–348
			Reverse	5′-CCCAAAGGTAAGAGTCACGAC-3′	429–449
Gal	Galanin	NM_010253.3	Forward	5′-GAGAGCAACATTGTCCGCAC-3′	396–415
			Reverse	5′-ATGGTCTCAGGACTTCTCTAGG-3′	505–527
Glul	Glutamate-ammonia ligase	NM_008131.3	Forward	5′-TGAGAGAACCATCCTATTCACTG-3′	1927–1949
			Reverse	5′-TAAGCAGTAATGAAGCTGAGACC-3′	2045–2067
Igfbp3	Insulin-like growth factor binding protein 3	NM_008343.2	Forward	5′-GGAAACCCATAACCGAGTGAC-3′	1475–1495
			Reverse	5′-TCCCACAGTTCCCAAGTAGATC-3′	1585–1606
Npy1r	Neuropeptide Y receptor 1	NM_010934.4	Forward	GCGACTCAGAGCATTTCTAAC	2547–2567
			Reverse	GCAGTGAAGGATAAATGGTGAG	2637–2658
Sstr1	Somatostatin receptor 1	NM_009216.3	Forward	5′-GCTGTCCAATTGAGTATGCTGC-3′	3419–3440
			Reverse	5′-CAGGTCAGTGTGAACTTGCG-3′	3527–3546
Ywhaz	Tyrosine 3-monooxygenase/tryptophan 5-monooxygenase activation protein, zeta polypeptide	NM_011740.3	Forward	5′-TCCTTATTCCCTCTTGGCAG-3′	2432–2451
			Reverse	5′-ATGGAAGCTACATTAGCGGTTT-3′	2502–2523

* CycA primer sequences were described in published literature [74].

## Materials and Methods

### Animals

Untested nulliparous female mice from a line of mice previously selected for high maternal defense (original stock was outbred hsd:ICR mice) (*Mus domesticus*) (Harlan, Madison, WI) were used. Selected mice were used because they exhibit the reliable emergence of a number of maternal characteristics, including nursing and offspring protection, and thus provide a solid platform for comparing maternal and non-maternal brains. As for any work that uses one strain, there is a limitation in knowing whether results apply to other strains. Only with comparisons across strains and species can reliable alterations with the postpartum period be identified, but here we provide a start for examining septal changes in gene expression with maternal behavior in mice. All animals were age matched (∼70 days old at time of dissection). For mating, females were housed with a breeder male (hsd:ICR strain) for 2 weeks. At the same time, virgin females were co-housed to provide similar social stimuli. When breeder males were removed, all females (pregnant and virgin) were housed individually and provided precut nesting material until dissections. Thus, virgin and postpartum females experienced similar levels of co-housing and single housing. The timing of cohousing and isolation was performed to minimize the effects of isolation-induced stress. Polypropylene cages were changed once weekly, but when pups were born (postpartum Day 0), cages were not changed for the postpartum female or the age matched virgin control for the remainder of the experiment. All animals were housed in the same room and cages of virgin and lactating females were alternated with one another on the same shelves. A 14∶10 light/dark cycle with lights on at 06∶00 h CST was used. Female mice were given ad lib access to breeder chow (Harlan) and tap water. All procedures were carried out in strict accordance with the guidelines of the National Institutes of Health Guide for the Care and Use of Laboratory Animals, and studies were approved by the University of Wisconsin Animal Care and Use Committee.

### Tissue Collection and RNA Extraction

On postpartum Days 6–8, brains were removed from lactating females between 10∶00 and 12∶00 h. Brains from age-matched, virgin females were also removed on the same day and dissections were alternated between the two groups so that an equal number of dissections from each group were obtained. The animals were lightly anaesthetized with isoflurane, killed by cervical dislocation and then decapitated. Following decapitation, virgin females were examined for stage of estrous cycle using a vaginal lavage [65], [66]. Female mice in vaginal proestrus were not used because this stage is associated with mating in mice. For the microarray study, the stages of the six virgin mice were estrous, diestrous I, and diestrous II (N = 2 each). Although estrous stage can influence gene expression [57], the use of mice from different estrous states (excluding proestrus) provides a generic view of the virgin female and should provide insights into reliable differences between maternal and virgin mice. The whole brain was removed, snap frozen in isopentane on dry ice, and then stored at −80°C until sliced. Brain sections were sliced in a cryostat (Leica, CM1850, Bannockburn, IL, USA) with the first two sections at 300 microns and the last section at 200 microns and then mounted on glass slides. Target tissue was captured using a micropunch technique [67]. Microdissection of frozen brain sections was made with Brain Punch Set (Stoelting, Wood Dale, IL, USA) under a dissecting microscope. The whole septum including lateral and medial parts (Fig. 2) was collected bilaterally from Bregma 1.10 to 0.14 and pooled, so that each mouse provided one sample of the septum. Microdissections from ten animals in each group were flash frozen on dry ice and stored at −80°C until being processed for either gene array analysis or real-time PCR. Total RNA was extracted and prepared in matched pairs (virgin versus lactating) using an Aurum Total RNA Fatty and Fibrous Tissue Kit (Bio-Rad, Hercules, CA, USA) according to the manufacturer’s instructions. Following isolation, the integrity of RNA was assessed using Agilent RNA 6000 Nano Chips with Agilent Bioanalyzer 2100 (Agilent Technologies, Palo Alto, CA). The purity of RNA was tested, and the yield of RNA was determined using NanoDrop 1000 spectrophotometer (Thermo Scientific, Wilmington, DE, USA). Purified total RNA was stored at −80°C until processed.

### High-Density Oligonucleotide Array Hybridization

Six out of ten samples in each group (n = 6 per group) were randomly chosen for use in the microarray experiment. Microarray analysis was performed with the GeneChip Mouse Gene 1.0 ST array (Affymetrix, Santa Clara, CA, USA) using targets derived from total RNA isolated from septum as described above. cDNA for array hybridization was synthesized from 200 ng of total RNA using an Ambion GeneChip WT Expression Kit (Ambion, Austin, TX, USA) according to the manufacturer’s specifications. Briefly, total RNA was used to synthesize double-stranded cDNA, which was then used as a template for single-stranded cRNA synthesis. This cRNA was in turn used as a template for a second round of single-stranded cDNA synthesis, and the resultant DNA-RNA hybrids were then degraded using RNase H. Amplified cDNA was then fragmented and biotinylated using an Affymetrix WT Terminal Labeling Kit (Affymetrix, Santa Clara, CA, USA) according to the manufacturer’s specifications. Fragmented and labeled cDNA samples were hybridized with the arrays at 45°C for 16 hours. The hybridized arrays were then washed and stained according to manufacturer specifications, and arrays were scanned at 570 nm on an Affymetrix GC3000 G7 Scanner. Data were extracted and processed from scans using Affymetrix Command Console v. 3.1.1.1229. cDNA synthesis, fragmentation, labeling, array hybridization, staining, and scanning were performed by the Gene Expression Center at the University of Wisconsin–Madison.

### Probeset Level Summarization and Microarray Statistical Analysis

Probeset level summarization and data normalization were performed with the PLIER algorithm with GC bin background correction using Affymetrix Power Tools v. 1.12.0. The microarray data discussed in this publication, both raw and summarized, have been deposited in NCBI’s Gene Expression Omnibus [68], and are accessible through GEO Series accession number GSE30836 (http://www.ncbi.nlm.nih.gov/geo/query/acc.cgi?acc=GSE30836). GEO reporting fulfills the requirements of the MIAME. Inferential statistics for differential expression between maternal and virgin samples were calculated using the array-specific empirical Bayesian implementation of ANOVA in the BioConductor package limma v.3.6.9 [69]. Both nominal and false discovery rate corrected p-values were calculated, and fold-change differences for each gene were calculated in Excel by dividing the limma-calculated average maternal expression by the limma-calculated average virgin expression.

### Verification of Microarray Results with Quantitative Real-time PCR (qPCR)

To confirm gene expression results obtained from microarray analysis, six genes: fatty acid binding protein 7 (Fabp7), galanin (Gal), glutamate-ammonia ligase (Glul)/glutamine synthetase (GS), insulin-like growth factor binding protein 3 (Igfbp3), neuropeptide Y receptor 1 (Npy1r), and somatostatin receptor 1 (Sstr1) were evaluated using qPCR. These genes were chosen because of their possible involvement in multiple lactation-induced processes and the function of the genes is described in the Discussion. A SuperScript III First-Strand Synthesis System for RT-PCR (Invitrogen, Carlsbad, CA, USA) was used to reverse transcribe 100 ng of RNA to cDNA in an Eppendorf MasterCycler Personal PCR machine (Eppendorf, Hamburg, Germany) using poly-T 20mer primers. The cDNA was then amplified using a SsoFast EvaGreen Supermix kit (Bio-Rad, Hercules, CA, USA) in a StepOnePlus real-time PCR system (Applied Biosystems, Foster City, CA, USA). The amplification mixtures (20 µL) contained 1x SsoFast EvaGreen Supermix, 160 ng template cDNA, and 500 nM forward and reverse primers. Each sample was run in triplicate and standard amplification procedures were used. The cycling profile is as follows: an initial melting step at 95°C for 30 Sec followed by 40 cycles of a 95°C melting step for 5 sec, a 58°C annealing step except Fabp7 (at 60°C) for 20 sec, and a 72°C elongation step for 20 sec. Primers for genes of interest and reference genes (Table 4) were designed and screened for specificity using NCBI Primer-BLAST. Ywhaz and CycA were used as reference genes, as they are found to be among the most stable genes in rodent brain [70], [71] and found not to be different between lactating and virgin females in our arrays results. Following amplification, a standard curve was generated to assess the empirical PCR reaction efficiency, and a dissociation curve analysis was performed to insure specificity of PCR products. C_t_ values were calculated using the StepOnePlus software. The expression ratio of mRNA of genes in lactating relative to virgin (normalized to the reference genes Ywhaz and CycA) was calculated using a relative expression software tool REST 2009, which corrects for empirical PCR efficiency, allows for the use of multiple reference genes, and utilizes a randomization test of significance [72]. The N’s used for qPCR were as follows: virgin, N = 10; maternal, N = 10. Four females were added to each group from the array mice and the virgin states were estrous (N = 2), diestrous I (N = 4), and diestrous II (N = 4).

### Gene Set Enrichment Analysis (GSEA)

GSEA is a technique that calculates up or down regulation of precompiled lists of genes and was performed using GSEA software v. 2.07 [30]. The specific gene sets described here came from the MSigDB C3 computationally-curated gene set of miRNA and transcription factor binding sites. For transcription factor binding sites, data from multiple species were used, including mice, humans, and rats. For miRNA, only human data sets were available, but binding sites for given miRNAs in mice and humans are highly conserved [73]. The analysis used z-transformed values for PLIER-normalized array intensities, t-tests to calculate individual gene inferential statistics, gene set permutation, and a weighted test statistic. The GSEA output provides both nominal and corrected p-values for each target.

## Supporting Information

Table S1 **Full list of all targets, their relative expression and** ***P*** **value ranking using PLIER algorithm.**
(XLS)

Table S2 **List of target sites linked significantly with upregulation of subsets of genes in postpartum mice by GSEA.**
(DOC)
