# Supplementary material for: Gene Expression Changes in the Septum: Possible Implications for MicroRNAs in Sculpting the Maternal Brain
Source: PLoS One. 2012 Jun 6;7(6):e38602. doi: 10.1371/journal.pone.0038602 (PMC3368935; doi:10.1371/journal.pone.0038602)
Supplement: Table S2 — List of target sites linked significantly with upregulation of subsets of genes in postpartum mice by GSEA. (DOC) [file pone.0038602.s002.doc]

**Supplementary Table 2**

List of target sites linked significantly with upregulation of subsets of genes in postpartum mice by GSEA. Lists of target genes for each identifier are found at GSEA website (http://www.broadinstitute.org/gsea/index.jsp).

| **Rank** | **GSEA Identifier** | |  |  |  | **nominal p-value** | **FDR q-value** | **FWER p-value** |
| --- | --- | --- | --- | --- | --- | --- | --- | --- |
| 1 | V$ELK1_02 | |  |  |  | 0.0000 | 0.0000 | 0.0000 |
| 2 | TGCGCANK_UNKNOWN | | |  |  | 0.0000 | 0.0000 | 0.0000 |
| 3 | GCCATNTTG_V$YY1_Q6 | | |  |  | 0.0000 | 0.0000 | 0.0000 |
| 4 | ACTAYRNNNCCCR_UNKNOWN | | |  |  | 0.0000 | 0.0000 | 0.0000 |
| 5 | GTGCCAT,MIR-183 | |  |  |  | 0.0000 | 0.0000 | 0.0000 |
| 6 | ATGTTAA,MIR-302C | |  |  |  | 0.0000 | 0.0000 | 0.0000 |
| 7 | GTACTGT,MIR-101 | |  |  |  | 0.0000 | 0.0000 | 0.0000 |
| 8 | V$NRF2_01 | |  |  |  | 0.0000 | 0.0000 | 0.0000 |
| 9 | TGAATGT,MIR-181A,MIR-181B,MIR-181C,MIR-181D | | | | | 0.0000 | 0.0000 | 0.0000 |
| 10 | V$YY1_02 |  |  |  |  | 0.0000 | 0.0000 | 0.0000 |
| 11 | AGCACTT,MIR-93,MIR-302A,MIR-302B,MIR-302C,MIR-302D,MIR-372,MIR-373,MIR-520E,MIR-520A,MIR-526B,MIR-520B,MIR-520C,MIR-520D | | | | | 0.0000 | 0.0000 | 0.0000 |
| 12 | V$CREBP1_01 | |  |  |  | 0.0000 | 0.0000 | 0.0000 |
| 13 | CAGTATT,MIR-200B,MIR-200C,MIR-429 | | | |  | 0.0000 | 0.0000 | 0.0000 |
| 14 | TTGCACT,MIR-130A,MIR-301,MIR-130B | | | |  | 0.0000 | 0.0000 | 0.0000 |
| 15 | TCCAGAG,MIR-518C | |  |  |  | 0.0000 | 0.0000 | 0.0000 |
| 16 | ACATTCC,MIR-1,MIR-206 | | |  |  | 0.0000 | 0.0000 | 0.0000 |
| 17 | CAGTGTT,MIR-141,MIR-200A | | |  |  | 0.0000 | 0.0000 | 0.0000 |
| 18 | GCATTTG,MIR-105 | |  |  |  | 0.0000 | 0.0000 | 0.0000 |
| 19 | TTTGCAC,MIR-19A,MIR-19B | | |  |  | 0.0000 | 0.0000 | 0.0000 |
| 20 | GTGACGY_V$E4F1_Q6 | | |  |  | 0.0000 | 0.0000 | 0.0000 |
| 21 | CAGCTTT,MIR-320 | |  |  |  | 0.0000 | 0.0000 | 0.0000 |
| 22 | ACTGCAG,MIR-17-3P | |  |  |  | 0.0000 | 0.0000 | 0.0000 |
| 23 | GTTTGTT,MIR-495 | |  |  |  | 0.0000 | 0.0000 | 0.0000 |
| 24 | TTCNRGNNNNTTC_V$HSF_Q6 | | |  |  | 0.0000 | 0.0000 | 0.0000 |
| 25 | GAGACTG,MIR-452 | |  |  |  | 0.0000 | 0.0000 | 0.0000 |
| 26 | GCACTTT,MIR-17-5P,MIR-20A,MIR-106A,MIR-106B,MIR-20B,MIR-519D | | | | | 0.0000 | 0.0000 | 0.0000 |
| 27 | ATGTACA,MIR-493 | |  |  |  | 0.0000 | 0.0000 | 0.0000 |
| 28 | GKCGCNNNNNNNTGAYG_UNKNOWN | | | |  | 0.0000 | 0.0000 | 0.0000 |
| 29 | GCGNNANTTCC_UNKNOWN | | |  |  | 0.0000 | 0.0000 | 0.0000 |
| 30 | TTAYRTAA_V$E4BP4_01 | | |  |  | 0.0000 | 0.0000 | 0.0000 |
| 31 | CAGCCTC,MIR-485-5P | | |  |  | 0.0000 | 0.0000 | 0.0000 |
| 32 | GGCNKCCATNK_UNKNOWN | | |  |  | 0.0000 | 0.0000 | 0.0000 |
| 33 | TTGCCAA,MIR-182 | |  |  |  | 0.0000 | 0.0000 | 0.0000 |
| 34 | ATTCTTT,MIR-186 | |  |  |  | 0.0000 | 0.0000 | 0.0000 |
| 35 | GACAATC,MIR-219 | |  |  |  | 0.0000 | 0.0000 | 0.0000 |
| 36 | CAATGCA,MIR-33 | |  |  |  | 0.0000 | 0.0000 | 0.0000 |
| 37 | GTATGAT,MIR-154,MIR-487 | | |  |  | 0.0000 | 0.0000 | 0.0000 |
| 38 | TGTTTAC,MIR-30A-5P,MIR-30C,MIR-30D,MIR-30B,MIR-30E-5P | | | | | 0.0000 | 0.0000 | 0.0000 |
| 39 | CACTGTG,MIR-128A,MIR-128B | | |  |  | 0.0000 | 0.0000 | 0.0000 |
| 40 | AGGGCAG,MIR-18A | |  |  |  | 0.0000 | 0.0000 | 0.0000 |
| 41 | TGAGATT,MIR-216 | |  |  |  | 0.0000 | 0.0000 | 0.0000 |
| 42 | TAGCTTT,MIR-9 | |  |  |  | 0.0000 | 0.0000 | 0.0000 |
| 43 | CTTTGTA,MIR-524 | |  |  |  | 0.0000 | 0.0000 | 0.0000 |
| 44 | V$YY1_Q6 | |  |  |  | 0.0000 | 0.0000 | 0.0000 |
| 45 | V$NFMUE1_Q6 | |  |  |  | 0.0000 | 0.0000 | 0.0000 |
| 46 | GTGCCAA,MIR-96 | |  |  |  | 0.0000 | 0.0000 | 0.0000 |
| 47 | CTTTGCA,MIR-527 | |  |  |  | 0.0000 | 0.0000 | 0.0000 |
| 48 | CACTGCC,MIR-34A,MIR-34C,MIR-449 | | | |  | 0.0000 | 0.0000 | 0.0000 |
| 49 | ACTTTAT,MIR-142-5P | | |  |  | 0.0000 | 0.0000 | 0.0000 |
| 50 | TACTTGA,MIR-26A,MIR-26B | | |  |  | 0.0000 | 0.0000 | 0.0000 |
| 51 | AACATTC,MIR-409-3P | | |  |  | 0.0000 | 0.0000 | 0.0000 |
| 52 | ACACTGG,MIR-199A,MIR-199B | | |  |  | 0.0000 | 0.0000 | 0.0000 |
| 53 | GGCNNMSMYNTTG_UNKNOWN | | | |  | 0.0000 | 0.0000 | 0.0000 |
| 54 | TGCACTG,MIR-148A,MIR-152,MIR-148B | | | |  | 0.0000 | 0.0000 | 0.0000 |
| 55 | CTCAAGA,MIR-526B | |  |  |  | 0.0000 | 0.0000 | 0.0000 |
| 56 | AAGCACT,MIR-520F | |  |  |  | 0.0000 | 0.0000 | 0.0000 |
| 57 | GGCAGCT,MIR-22 | |  |  |  | 0.0000 | 0.0000 | 0.0000 |
| 58 | V$NRF1_Q6 | |  |  |  | 0.0000 | 0.0000 | 0.0000 |
| 59 | V$RFX1_02 | |  |  |  | 0.0000 | 0.0000 | 0.0000 |
| 60 | TNCATNTCCYR_UNKNOWN | | |  |  | 0.0000 | 0.0000 | 0.0000 |
| 61 | V$E4BP4_01 | |  |  |  | 0.0000 | 0.0000 | 0.0000 |
| 62 | AAGCACA,MIR-218 | |  |  |  | 0.0000 | 0.0000 | 0.0010 |
| 63 | GGTGTGT,MIR-329 | |  |  |  | 0.0000 | 0.0000 | 0.0010 |
| 64 | TGCCTTA,MIR-124A | |  |  |  | 0.0000 | 0.0000 | 0.0010 |
| 65 | GTGTTGA,MIR-505 | |  |  |  | 0.0000 | 0.0000 | 0.0010 |
| 66 | V$GABP_B | |  |  |  | 0.0000 | 0.0000 | 0.0010 |
| 67 | ACTGTAG,MIR-139 | |  |  |  | 0.0000 | 0.0000 | 0.0010 |
| 68 | CTCAGGG,MIR-125B,MIR-125A | | |  |  | 0.0000 | 0.0000 | 0.0010 |
| 69 | CCGNMNNTNACG_UNKNOWN | | |  |  | 0.0000 | 0.0000 | 0.0010 |
| 70 | GGAANCGGAANY_UNKNOWN | | |  |  | 0.0000 | 0.0000 | 0.0010 |
| 71 | TGTATGA,MIR-485-3P | | |  |  | 0.0000 | 0.0000 | 0.0010 |
| 72 | CATTTCA,MIR-203 | |  |  |  | 0.0000 | 0.0000 | 0.0010 |
| 73 | GGCAGTG,MIR-324-3P | | |  |  | 0.0000 | 0.0000 | 0.0010 |
| 74 | ATACCTC,MIR-202 | |  |  |  | 0.0000 | 0.0000 | 0.0010 |
| 75 | TTCYRGAA_UNKNOWN | | |  |  | 0.0000 | 0.0000 | 0.0010 |
| 76 | TCATCTC,MIR-143 | |  |  |  | 0.0000 | 0.0000 | 0.0010 |
| 77 | CTATGCA,MIR-153 | |  |  |  | 0.0000 | 0.0000 | 0.0010 |
| 78 | V$E2F_03 |  |  |  |  | 0.0000 | 0.0000 | 0.0010 |
| 79 | GTAAACC,MIR-299-5P | | |  |  | 0.0000 | 0.0000 | 0.0010 |
| 80 | ATATGCA,MIR-448 | |  |  |  | 0.0000 | 0.0000 | 0.0020 |
| 81 | ACTGTGA,MIR-27A,MIR-27B | | |  |  | 0.0000 | 0.0000 | 0.0020 |
| 82 | TTTGTAG,MIR-520D | |  |  |  | 0.0000 | 0.0000 | 0.0020 |
| 83 | CCTGCTG,MIR-214 | |  |  |  | 0.0000 | 0.0000 | 0.0020 |
| 84 | GGAMTNNNNNTCCY_UNKNOWN | | | |  | 0.0000 | 0.0000 | 0.0020 |
| 85 | TGTGTGA,MIR-377 | |  |  |  | 0.0000 | 0.0000 | 0.0020 |
| 86 | TGCTGCT,MIR-15A,MIR-16,MIR-15B,MIR-195,MIR-424,MIR-497 | | | | | 0.0000 | 0.0000 | 0.0020 |
| 87 | AACTGGA,MIR-145 | |  |  |  | 0.0000 | 0.0000 | 0.0020 |
| 88 | ACACTCC,MIR-122A | |  |  |  | 0.0000 | 0.0000 | 0.0020 |
| 89 | GTTRYCATRR_UNKNOWN | | |  |  | 0.0000 | 0.0000 | 0.0020 |
| 90 | GACTGTT,MIR-212,MIR-132 | | |  |  | 0.0000 | 0.0000 | 0.0020 |
| 91 | RGAANNTTC_V$HSF1_01 | | |  |  | 0.0000 | 0.0000 | 0.0020 |
| 92 | V$CETS1P54_01 | |  |  |  | 0.0000 | 0.0000 | 0.0020 |
| 93 | GAGCCAG,MIR-149 | |  |  |  | 0.0000 | 0.0000 | 0.0020 |
| 94 | GGCAGAC,MIR-346 | |  |  |  | 0.0000 | 0.0000 | 0.0020 |
| 95 | V$HIF1_Q3 | |  |  |  | 0.0000 | 0.0000 | 0.0020 |
| 96 | ACCAAAG,MIR-9 | |  |  |  | 0.0000 | 0.0000 | 0.0030 |
| 97 | GCAAAAA,MIR-129 | |  |  |  | 0.0000 | 0.0000 | 0.0030 |
| 98 | GTGCAAT,MIR-25,MIR-32,MIR-92,MIR-363,MIR-367 | | | | | 0.0000 | 0.0000 | 0.0040 |
| 99 | CTGTTAC,MIR-194 | |  |  |  | 0.0000 | 0.0000 | 0.0040 |
| 100 | V$ATF4_Q2 | |  |  |  | 0.0000 | 0.0000 | 0.0040 |
| 101 | AACTGAC,MIR-223 | |  |  |  | 0.0000 | 0.0000 | 0.0050 |
| 102 | CACCAGC,MIR-138 | |  |  |  | 0.0000 | 0.0000 | 0.0050 |
| 103 | ATGAAGG,MIR-205 | |  |  |  | 0.0000 | 0.0000 | 0.0050 |
| 104 | KCCGNSWTTT_UNKNOWN | | |  |  | 0.0000 | 0.0000 | 0.0050 |
| 105 | CACGTTT,MIR-302A | |  |  |  | 0.0000 | 0.0000 | 0.0050 |
| 106 | GAGCTGG,MIR-337 | |  |  |  | 0.0000 | 0.0000 | 0.0050 |
| 107 | TAANNYSGCG_UNKNOWN | | |  |  | 0.0000 | 0.0000 | 0.0050 |
| 108 | AAAGGGA,MIR-204,MIR-211 | | |  |  | 0.0000 | 0.0000 | 0.0050 |
| 109 | TTTTGAG,MIR-373 | |  |  |  | 0.0000 | 0.0000 | 0.0060 |
| 110 | GTGACTT,MIR-224 | |  |  |  | 0.0000 | 0.0000 | 0.0060 |
| 111 | GCTCTTG,MIR-335 | |  |  |  | 0.0000 | 0.0000 | 0.0060 |
| 112 | ACTGAAA,MIR-30A-3P,MIR-30E-3P | | | |  | 0.0000 | 0.0000 | 0.0060 |
| 113 | ATAAGCT,MIR-21 | |  |  |  | 0.0000 | 0.0000 | 0.0060 |
| 114 | AGCTCCT,MIR-28 | |  |  |  | 0.0000 | 0.0000 | 0.0060 |
| 115 | SGCGSSAAA_V$E2F1DP2_01 | | |  |  | 0.0000 | 0.0000 | 0.0060 |
| 116 | CAGCACT,MIR-512-3P | | |  |  | 0.0000 | 0.0000 | 0.0070 |
| 117 | TCANNTGAY_V$SREBP1_01 | | |  |  | 0.0000 | 0.0000 | 0.0070 |
| 118 | TGCACTT,MIR-519C,MIR-519B,MIR-519A | | | |  | 0.0000 | 0.0000 | 0.0070 |
| 119 | TTTGCAG,MIR-518A-2 | | |  |  | 0.0000 | 0.0000 | 0.0070 |
| 120 | TGCAAAC,MIR-452 | |  |  |  | 0.0000 | 0.0000 | 0.0080 |
| 121 | V$MYCMAX_B | |  |  |  | 0.0000 | 0.0000 | 0.0080 |
| 122 | V$SOX9_B1 | |  |  |  | 0.0000 | 0.0000 | 0.0080 |
| 123 | V$HLF_01 |  |  |  |  | 0.0000 | 0.0000 | 0.0080 |
| 124 | GCTGAGT,MIR-512-5P | | |  |  | 0.0000 | 0.0000 | 0.0080 |
| 125 | V$SF1_Q6 | |  |  |  | 0.0000 | 0.0000 | 0.0080 |
| 126 | V$ZF5_01 |  |  |  |  | 0.0000 | 0.0000 | 0.0090 |
| 127 | YYCATTCAWW_UNKNOWN | | |  |  | 0.0000 | 0.0000 | 0.0090 |
| 128 | ATCTTGC,MIR-31 | |  |  |  | 0.0000 | 0.0000 | 0.0090 |
| 129 | ACTGCCT,MIR-34B | |  |  |  | 0.0000 | 0.0000 | 0.0110 |
| 130 | V$HIF1_Q5 | |  |  |  | 0.0000 | 0.0000 | 0.0110 |
| 131 | GTTGNYNNRGNAAC_UNKNOWN | | | |  | 0.0000 | 0.0000 | 0.0110 |
| 132 | CACTTTG,MIR-520G,MIR-520H | | |  |  | 0.0000 | 0.0000 | 0.0110 |
| 133 | TAATGTG,MIR-323 | |  |  |  | 0.0000 | 0.0000 | 0.0120 |
| 134 | V$HSF2_01 | |  |  |  | 0.0000 | 0.0000 | 0.0130 |
| 135 | GTTATAT,MIR-410 | |  |  |  | 0.0000 | 0.0000 | 0.0130 |
| 136 | V$NMYC_01 | |  |  |  | 0.0000 | 0.0000 | 0.0130 |
| 137 | ATACTGT,MIR-144 | |  |  |  | 0.0000 | 0.0000 | 0.0130 |
| 138 | ATGTTTC,MIR-494 | |  |  |  | 0.0000 | 0.0000 | 0.0130 |
| 139 | TCCCRNNRTGC_UNKNOWN | | |  |  | 0.0000 | 0.0000 | 0.0130 |
| 140 | AAAGGAT,MIR-501 | |  |  |  | 0.0000 | 0.0000 | 0.0140 |
| 141 | GTATTAT,MIR-369-3P | | |  |  | 0.0000 | 0.0000 | 0.0140 |
| 142 | ATGCTGC,MIR-103,MIR-107 | | |  |  | 0.0000 | 0.0000 | 0.0140 |
| 143 | CATGTAA,MIR-496 | |  |  |  | 0.0000 | 0.0000 | 0.0140 |
| 144 | V$HSF1_01 | |  |  |  | 0.0000 | 0.0000 | 0.0140 |
| 145 | ATAGGAA,MIR-202 | |  |  |  | 0.0000 | 0.0000 | 0.0140 |
| 146 | RRAGTTGT_UNKNOWN | | |  |  | 0.0000 | 0.0000 | 0.0140 |
| 147 | TCCATTKW_UNKNOWN | | |  |  | 0.0000 | 0.0000 | 0.0140 |
| 148 | MCAATNNNNNGCG_UNKNOWN | | | |  | 0.0000 | 0.0000 | 0.0150 |
| 149 | AGGCACT,MIR-515-3P | | |  |  | 0.0000 | 0.0000 | 0.0150 |
| 150 | GTCAGGA,MIR-378 | |  |  |  | 0.0000 | 0.0000 | 0.0170 |
| 151 | V$ATF_01 |  |  |  |  | 0.0000 | 0.0000 | 0.0170 |
| 152 | V$E2F1DP1_01 | |  |  |  | 0.0000 | 0.0000 | 0.0190 |
| 153 | ACACTAC,MIR-142-3P | | |  |  | 0.0000 | 0.0000 | 0.0190 |
| 154 | V$E2F1DP2_01 | |  |  |  | 0.0000 | 0.0000 | 0.0190 |
| 155 | KMCATNNWGGA_UNKNOWN | | |  |  | 0.0020 | 0.0000 | 0.0190 |
| 156 | V$CREBP1_Q2 | |  |  |  | 0.0000 | 0.0000 | 0.0190 |
| 157 | V$E2F4DP2_01 | |  |  |  | 0.0000 | 0.0000 | 0.0210 |
| 158 | V$E2F_02 |  |  |  |  | 0.0000 | 0.0000 | 0.0210 |
| 159 | V$ERR1_Q2 | |  |  |  | 0.0000 | 0.0000 | 0.0220 |
| 160 | CTGAGCC,MIR-24 | |  |  |  | 0.0000 | 0.0000 | 0.0220 |
| 161 | TGACCTTG_V$SF1_Q6 | | |  |  | 0.0000 | 0.0000 | 0.0230 |
| 162 | ATGTAGC,MIR-221,MIR-222 | | |  |  | 0.0000 | 0.0000 | 0.0230 |
| 163 | ACAACTT,MIR-382 | |  |  |  | 0.0000 | 0.0000 | 0.0230 |
| 164 | AAGTCCA,MIR-422B,MIR-422A | | |  |  | 0.0000 | 0.0000 | 0.0230 |
| 165 | V$WHN_B | |  |  |  | 0.0000 | 0.0000 | 0.0230 |
| 166 | GGTAACC,MIR-409-5P | | |  |  | 0.0000 | 0.0000 | 0.0240 |
| 167 | GTCTTCC,MIR-7 | |  |  |  | 0.0000 | 0.0000 | 0.0240 |
| 168 | GTGGTGA,MIR-197 | |  |  |  | 0.0000 | 0.0000 | 0.0240 |
| 169 | GGCNRNWCTTYS_UNKNOWN | | |  |  | 0.0000 | 0.0000 | 0.0240 |
| 170 | CTTGTAT,MIR-381 | |  |  |  | 0.0000 | 0.0000 | 0.0240 |
| 171 | V$HSF_Q6 | |  |  |  | 0.0000 | 0.0000 | 0.0250 |
| 172 | TATTATA,MIR-374 | |  |  |  | 0.0000 | 0.0000 | 0.0250 |
| 173 | CGTSACG_V$PAX3_B | |  |  |  | 0.0000 | 0.0000 | 0.0250 |
| 174 | MGGAAGTG_V$GABP_B | | |  |  | 0.0000 | 0.0000 | 0.0250 |
| 175 | CCCAGAG,MIR-326 | |  |  |  | 0.0000 | 0.0000 | 0.0260 |
| 176 | TCTGATA,MIR-361 | |  |  |  | 0.0000 | 0.0000 | 0.0330 |
| 177 | GGGCATT,MIR-365 | |  |  |  | 0.0000 | 0.0000 | 0.0330 |
| 178 | ATGGYGGA_UNKNOWN | | |  |  | 0.0000 | 0.0000 | 0.0340 |
| 179 | TAGAACC,MIR-182 | |  |  |  | 0.0000 | 0.0000 | 0.0340 |
| 180 | V$E2F_Q6_01 | |  |  |  | 0.0000 | 0.0000 | 0.0340 |
| 181 | V$E2F4DP1_01 | |  |  |  | 0.0000 | 0.0000 | 0.0390 |
| 182 | TTGGGAG,MIR-150 | |  |  |  | 0.0000 | 0.0000 | 0.0390 |
| 183 | TCCAGAT,MIR-516-5P | | |  |  | 0.0000 | 0.0000 | 0.0390 |
| 184 | AACYNNNNTTCCS_UNKNOWN | | |  |  | 0.0000 | 0.0000 | 0.0410 |
| 185 | CTCCAAG,MIR-432 | |  |  |  | 0.0000 | 0.0000 | 0.0410 |
| 186 | SNACANNNYSYAGA_UNKNOWN | | | |  | 0.0020 | 0.0000 | 0.0430 |
| 187 | AGCATTA,MIR-155 | |  |  |  | 0.0000 | 0.0010 | 0.0460 |
| 188 | GCACCTT,MIR-18A,MIR-18B | | |  |  | 0.0000 | 0.0010 | 0.0460 |
| 189 | YGCANTGCR_UNKNOWN | | |  |  | 0.0000 | 0.0010 | 0.0470 |
| 190 | YGTCCTTGR_UNKNOWN | | |  |  | 0.0000 | 0.0010 | 0.0480 |
